# Ovarian, uterine, and luteal vascular perfusions during follicular and luteal phases in the adult cyclic female rabbits with special orientation to their histological detection of hormone receptor

**DOI:** 10.1186/s12917-022-03390-6

**Published:** 2022-08-04

**Authors:** Elshymaa A. Abdelnaby, Noha A. E. Yasin, Yara S. Abouelela, Eman Rashad, Samer M. Daghash, Hossam R. El-Sherbiny

**Affiliations:** 1 Theriogenology Department, Faculty of Veterinary Medicine, Cairo University, Giza, 12211 Egypt; 2 Cytology and Histology Department, Faculty of Veterinary Medicine, Cairo University, Giza, Egypt; 3 Anatomy and Embryology Department, Faculty of Veterinary Medicine, Cairo University, Giza, Egypt

**Keywords:** Estrogen receptor, Endothelial growth factor, Doppler, Progesterone receptor, Rabbit, Ovarian artery, Vascular

## Abstract

Understanding the does reproductive hemodynamic changes during the estrous cycle is crucial for improving reproductive competence and fertility potential in this species. The objective of this study is to investigate the hemodynamic variations in ovarian (OA) and uterine (UA) arteries, histological and morphometric changes in ovarian and uterine tissues throughout the follicular (FP) and luteal (LP) phases in rabbits and determine estrogen (ER), progesterone (PR) receptors, and vascular endothelial growth factor (VEGF) distributions using immunohistochemistry.

Fourteen adults pluriparous New Zealand rabbits were divided into rabbits at the FP (Day − 1; *n* = 7) and those at the LP (Day 9; *n* = 7). Animals were subjected to Doppler, hormonal (estrogen [E2], progesterone [P4], insulin-like growth factor [ILGF], and VEGF), histological, and immunohistochemical analyses. In LP, OA Doppler indices were significantly increased, whereas peak systolic velocity (PSV) was decreased compared with that in FP. UA Doppler indices were significantly decreased in the LP, whereas PSV was increased (*P* < 0.05). E2 levels were increased in the FP, whereas P4 levels were increased in the LP. The morphometric analysis of uterine tissues during the LP revealed an increase in the mean uterine endometrium length, endometrial connective tissue area percentage (%), endometrial glands number, myometrial area (%) and thickness. Furthermore, ovarian follicles and corpus luteum (CL) displayed strong positive immunoreactivity for ER, PR, and VEGF-A during both phases. The ovarian sections displayed a substantial (*P* < 0.05) increase in the area % of VEGF-A in the ovarian follicles during FP while in the CL during LP. Conversely, area percentage of VEGF-A immunoreactivity in the uterine luminal and glandular epithelia during the FP and LP revealed no differences. However, the number of VEGF-A–stained blood capillaries revealed an increase during LP than FP. In conclusion, this study demonstrated for the first time the changes in both ovarian and uterine arteries during two different phases of the rabbit cycle in relation to the histo-morphometric analysis and distribution of ER, PR, and VEGF-A, which regulate uterine functions that play a role in reproduction.

## Background

Due to their friendliness, ease of handling and observation, and the potential for breeding, rabbits were a frequent species used as an animal models in scientific research in human and animal medicine [1–3]. Rabbits are induced ovulators; therefore, pregnancy is particularly distinct, which is just hours or days after mating [4, 5]. Receptive doe displays a larger number of ovarian follicles with an increased estrogen hormone level [6], resulting in reddish swollen vulva and lordosis [7]. After coitus, the luteinizing hormone (LH) is released and reaches its maximum levels 1 h later, which results in ovulation [8, 9]. Currently, the estrogen hormone is dominant until corpus luteum (CL) formation [10]. The rabbit ovary and uterus undergo substantial transformation following ovulation [4]. The endometrium undergoes morphological and physiological cyclic changes, including proliferation, differentiation, apoptosis, and regeneration [11, 12]. Therefore, angiogenesis, which is the development of new vasculature from preexistent vessels, plays a fundamental role in tissue remodeling and is necessary for implantation and normal pregnancy and proper uterine blood supply maintenance [13, 14].

During the reproductive cycle, both estrogen and progesterone hormones play a crucial role in controlling the uterine cyclic changes and functions of their receptors. Furthermore, these hormones influence the uterine vasculature growth either directly through their receptors or indirectly by initiating proangiogenic factor release, including vascular endothelial growth factor (VEGF) [12, 15]. VEGF is one of the most common angiogenic factors that stimulate robust angiogenesis, increases blood vessel permeability, and plays a crucial role in ovarian folliculogensis and consequently CL formation and maintenance [16–18]. VEGF exists in five isoforms, including VEGF-A, B, C, D, and E, and acts through three tyrosine kinase family receptors [16]. VEGF-A is the key mediator of the angiogenic process, and its activity is mediated by binding to VEGF receptors (VEGF-R1 and VEGF-R2) on endothelial cells [19]. By focusing its impact on (luteal phase) LP stages, VEGF protein is found in the hormone-producing cells of the CL in primates, with the largest concentration in granulosa-derived cells [20, 21]. VEGF is present throughout the LP; however, it becomes less prevalent as luteolysis progresses. Conversely, in sheep CL, a highly specific antibody detected VEGF in vascular pericytes although not in hormone-producing cells [22]. Moreover, ovarian vessel formation and functionality mainly depend on VEGF-A due to its role in angiogenesis [23]. Insulin-like growth factors (IGFs) are essential steroidogenesis promoters that could act at various points along the production route, such as promoting cholesterol substrate absorption, suppressing apoptosis that helps in luteal weight maintenance [24], and stimulating P4 secretion acutely. It was found that the members of the IGF family play significant roles in boosting angiogenesis through VEGF creation in luteal cells and steroid synthesis through major steroidogenic protein generation [25].

There are two main classical isoforms of estrogen receptor (ER): Erα and Erβ [26, 27]. ER and progesterone receptor (PR) were distinguished in the buffalo ovarian sections [28], both luminal and glandular epithelial cells in addition to stromal cells in the uterus of bovine [28] during the follicular (FP) and luteal (LP) phases and in rabbits during pseudopregnancy [29]. Nitric oxide (NO) is a paracrine mediator with a wide range of physiological roles, including arterial dilation/permeability modulation [30] and neurotransmission [31]. Several lines of evidence imply that NO is involved in cycle-dependent ovarian events, such as ovulation and luteal function modulation [32, 33]. To the best of our knowledge, the occurrence and distribution of these critical hormonal receptors (ER, PR, and VEGF-A) in the ovarian and uterine tissues with alterations in the vascularization of rabbits at the FP and LP have not been well studied. The practical applications of this study are summarized in the determination of the normal blood flow in both ovarian and luteal arteries in order to make a perfect judgment on the basic reproductive or biotechnological aspects of the rabbit. Therefore, this study aimed for the first time to investigate the hemodynamic variations in the ovarian (OA) and uterine (UA) arteries as well as the morphological and morphometric changes in the ovarian and uterine tissues during these two phases and determine the ER, PR, and VEGF-A distribution in these tissues in correlation to their serum hormonal changes using immunohistochemistry, as this study could open a wide field in rabbit reproduction via demonstration of the normal vascularization that occurred in those phases.

## Materials and methods

### Ethical statement

Ethical approval (VET CU 12/10/2021/385) for this study was provided by Institutional Animal Care and Use Committee of the Faculty of Veterinary Medicine, Cairo University.

### Animals and housing

This study was conducted at Cairo university, Faculty of Veterinary Medicine at the Departments of Theriogenology, Anatomy, and Histology. The current study was performed on 14 cyclic healthy pluriparous female New Zealand rabbits (weighed 4.5–5.5 kg, with an average of 5 ± 0.5 kg; aged 3–4 years, with an average of 3.5 ± 0.5 years). All animals were divided into two groups: rabbits at the FP (Day − 1; *n* = 7) that were previously synchronized by receiving a subcutaneous 25 IU injection of PMSG 48 hr. before mating and subsequently received 25 μg of GnRH (Gonadorelin, Fertagyl; Intervet Inc., Boxmeer, Netherlands) following 48 hr. of forced mating with a sterile adult male [34]; the first FP after synchronization was examined. Rabbits at the LP (Day 9; *n* = 7) comprised the second group, which were previously synchronized and subsequently mated with a sterile adult male. The animals were anesthetized and sacrificed for anatomical examination (*n* = 4) and histological examinations (*n* = 10). All rabbits received food and water ad libitum and were housed in cages.

### Ultrasound scanning and Doppler analysis

B-mode ultrasonography was performed after mating for 1 day to confirm ovulation, and this day was referred to as day 1 of the LP. Conversely, the FP was determined as the day before ovulation with the presence of the largest preovulatory follicle (Day − 1).

**Fig. 1:**
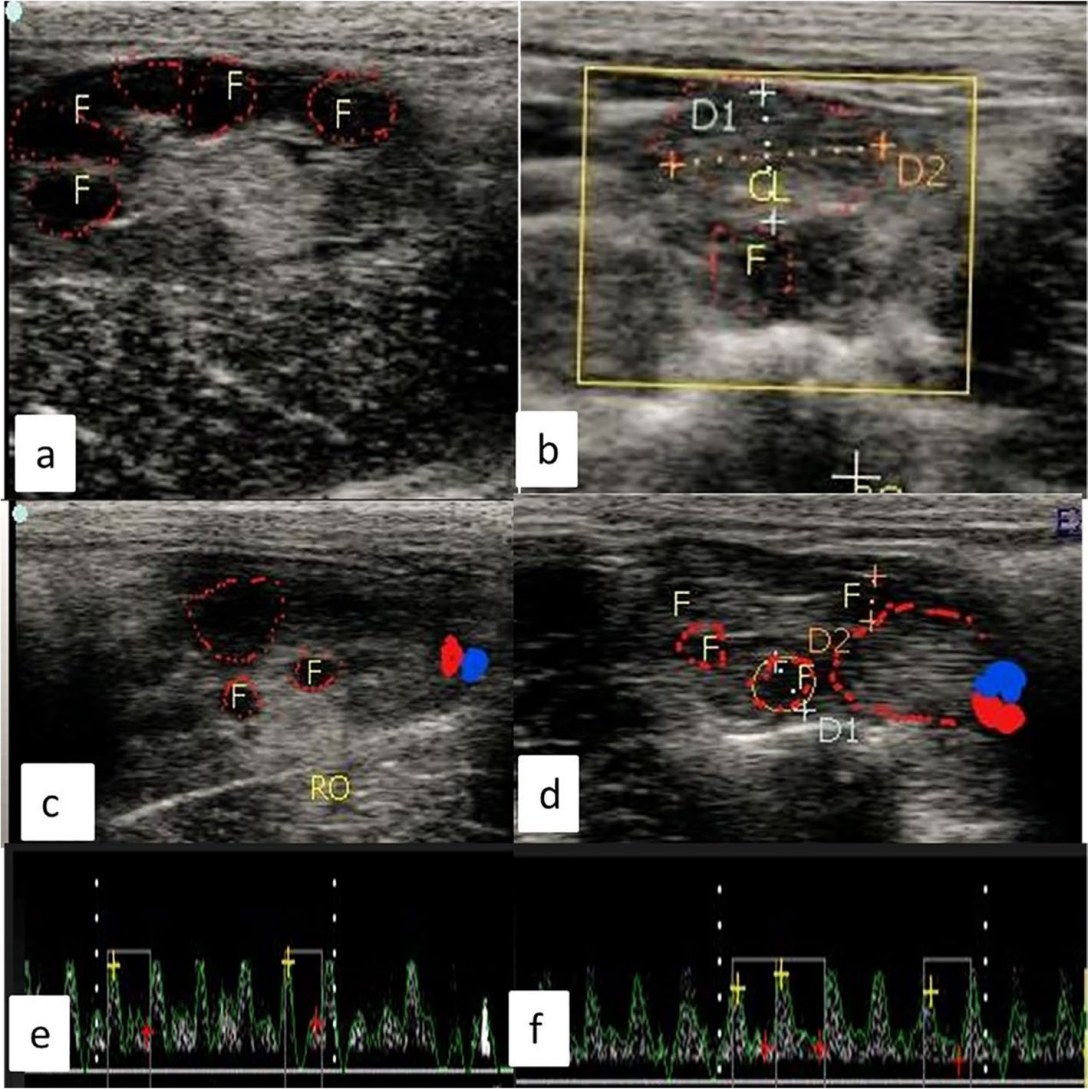
B-mode ultrasonograms revealed numerous follicles during the follicular phase at day − 3 (**a**) and corpus luteum during the luteal phase at day 9(**b**),and color mode ultrasonograms revealed ovarian artery coloration during the follicular phase at day − 1 with presence of largest preovulatory follicle(**c**),while in the luteal phase the color mode showed a luteal artery supplies the corpus luteum with presence of small growing follicles(**d**),(**e** and **f**) showed spectral graph of both ovarian and luteal artery in both follicular (day − 1) and luteal (day 9) phases in female rabbit. F = follicle, CL = corpus luteum, RO = right ovary

B-mode ultrasonogram (EXAGO, Meyreuil, France; brightness, 70%; depth, 3 cm; acoustic power, 87%; spectral insonation angle, 55°; and PRF, 3500 Hz) was performed using a 7.5-MHz, linear array probe. All examinations were performed by the same professional. B-mode ultrasonography of both ovaries and uterus was performed on days − 1 and 9. Also, B-mode ultrasonography was performed on day-3 to reveal the numerous follicles, as follicles on that day before ovulation are more than one, while with the progress of the ovulation the largest preovulatory one is ruptured which was confirmed by ultrasonography (Fig. 1a) and corpora lutea during the LP Day 9 (Fig. 1b). However, in color mode ultrasonograms, ovarian artery (OA) coloration was revealed during the FP with the presence of the largest preovulatory follicle (Fig. 1c), whereas a luteal artery (LA) supplying the CL with the presence of small growing follicles were observed in the LP (Fig. 1d).

**Fig. 2:**
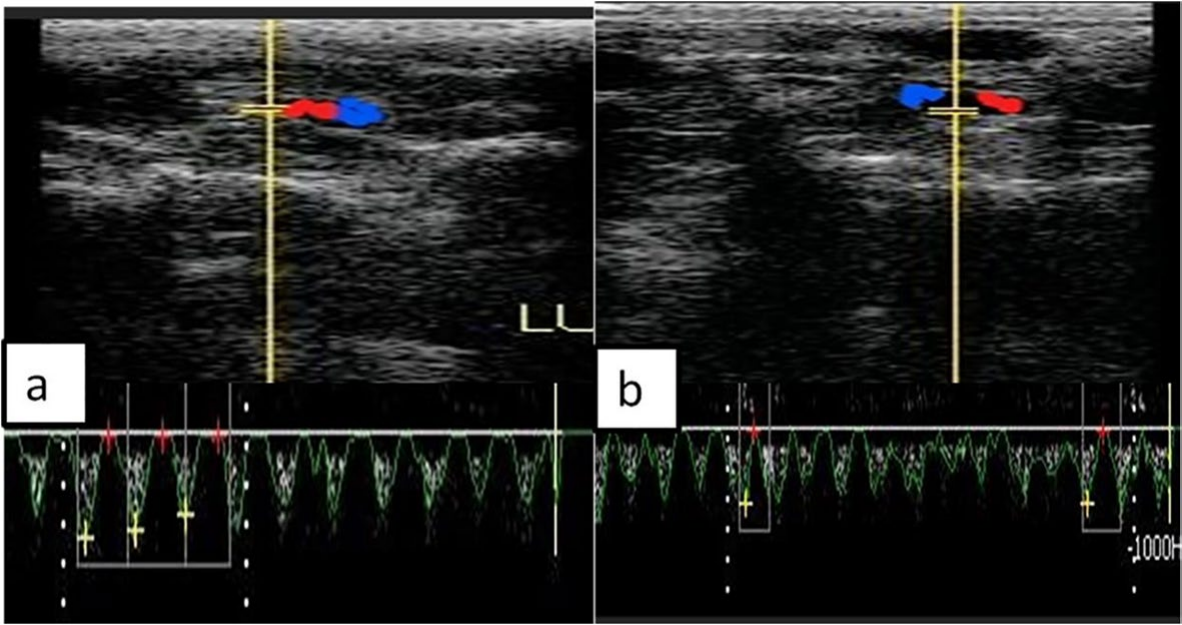
Ultrasonograms with color and spectral Doppler modes revealed cross-sectional coloration of the middle uterine artery during both follicular (day − 1) and luteal (day 9) phases in female rabbit

The spectral wave was utilized to calculate Doppler parameters, including resistance index (RI), pulsatility index (PI), and peak systolic velocity (PSV, cm/sec), to obtain the wave graph to evaluate the functionality of the known specific artery as ovarian (Fig. 1e, f), luteal, and uterine (Fig. 2a, b). In addition to uterine artery cross-sectional diameter was determined after color mode activation. RI was measured by an automatic equation in the device as follows RI = [PSV-EDV/PSV], while PI = [PSV-EDV/TAV], as TAV was the time average velocity to complete one cardiac cycle and EDV was the end point of velocity in the spectral graph as previously measured [35–37].

### Anatomical examination and arterial angioarchitecture

Rabbits were anaesthetized with xylazine (1 mg/kg, IM) and ketamine (5% IV 10 mg/kg); then, they were sacrificed by exsanguination of the carotid arteries that permit the releasing of blood from blood vessels to facilitate its injection with latex later on. The exposed abdominal aorta was cannulated and rinsed carefully from clotted blood using warm normal saline; a gum milk latex emulsion (60%) colored with red ROTRING ink was subsequently injected [38]. Then, the specimens were kept in a refrigerator until the latex was solidified and delight dissection occurred to examine the genital tract and its ovarian and uterine arterial supplies.

**Fig. 3:**
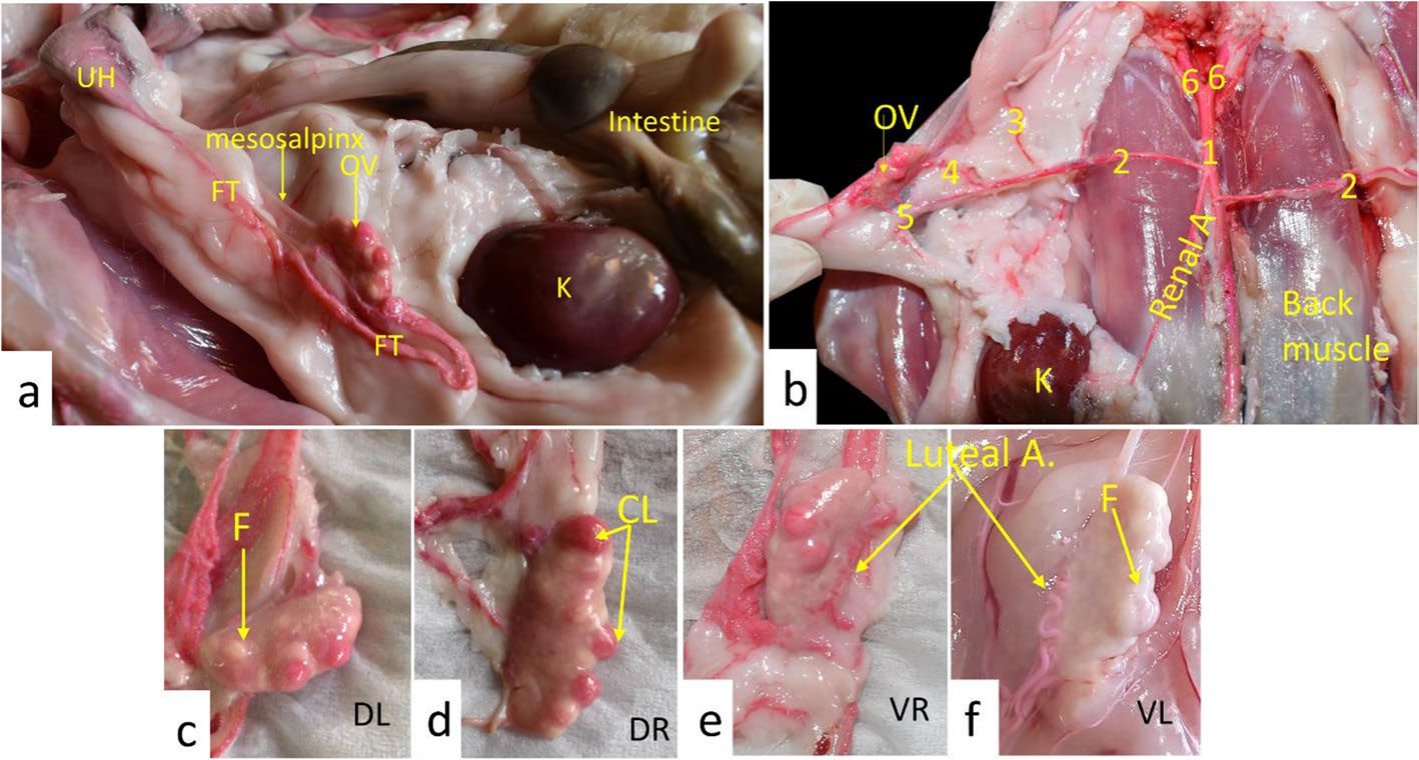
Anatomical pictures demonstrated the rabbit ovary insitu (**a**), the origin of the ovarian artery (**b**), DL (dorsal view of the left ovary; **c**), DR (dorsal view of the right ovary; **d**), VR (ventral view of the right ovary; **e**), and VL (Ventral view of the left ovary; **f**). OV = ovary, FT = fallopian tube, UH = uterine horn, K = kidney, CL = corpus luteum, F = follicle.1- Abdominal aorta, 2-Ovarian artery, 3- Uterine branch of ovarian artery, 4- Tubal branch of ovarian artery, 5- Ovarian branch of ovarian artery, and 6- Common iliac artery

Based on our anatomical findings, the New Zealand rabbit ovary was suspended in the lateral abdominal wall by mesovarian ligament just caudal to the kidneys on both sides. It was elliptical, compressed dorsoventrally, and carried several follicles and CL on its surface that gave it an irregular surface (Fig. 3).

### Blood sampling and hormonal analysis

Blood samples were collected in 2-mL syringes from the auricular vein and centrifuged at 2000×g for 10 min. Serum samples were stored at − 18 °C for hormonal assay. Estradiol 17β, progesterone, and IGF-1 were assayed using DRG diagnostics (Diagnostic Reagents) using ELISA with 9.8, 0.04, and 1.29 ng/mL assay sensitivities, respectively, as IGF-1 was analyzed using antibodies with high sensitivity for two different epitopes on IGF-1 [39]. IGF-1 was determined by ELISA kit with a catalogue reference number (eia-4140).VEGF serum levels were measured using an ELISA kit with a 2.2-pg/mL sensitivity at (SINULOG, China; catalogue number: SL2247Hu). NO is determined by serum samples [40].

### Histological investigation

#### General histological examination

Histological preparation procedures were conducted following the protocol described by [41]. Briefly, the ovarian and uterine tissues were sliced to 3–4-mm thickness, fixed in 10% neutral buffered formalin for 48 h, dehydrated in graded ethanol concentrations, cleared in xylene, and embedded in paraffin. To examine the general tissue structure, the paraffin blocks were sectioned via a rotatory microtome at 4–6-μm thickness and dyed with hematoxylin and eosin (H&E) stain. Photographs were captured under different powers using Leica microscope (CH9435, Hee56rbrugg) (Leica Microsystems, Switzerland).

#### Morphometric analysis

The endometrial length, area percentage (%) of the endometrial connective tissue (CT), number of endometrial glands as well as thickness and area % of the myometrium were assessed using the Image J program.

#### Immunohistochemistry

Thick deparaffinized ovarian and uterine sections (3–5 μm) were prepared for the immunohistochemical expression of ERα, PR, and VEGF-A following the manufacturer’s protocol. Slides were quenched in 3% hydrogen peroxide, washed in PBS, and blocked in 1% bovine serum albumin. Subsequently, they were incubated with a primary antibody monoclonal Anti-Mouse Estrogen Receptor alpha Monoclonal Antibody (EVG F9) (Thermo-Fisher Scientific, Cat# # MA3–310, RRID: AB_347010, Dilution: 1:200), Anti Mouse Progesterone Receptor Monoclonal Antibody (PR-AT 4.14) (Thermo-Fisher Scientific, Cat# MA1–410, RRID AB_2164327, Dilution: 5 μg/mL), and polyclonal anti-VEGF-A rabbit pAb (Thermo-Fisher Scientific, Cat# GB-14400, Dilution: 1:500–1:1000) for 1 h; the slides were washed out by PBS and immediately incubated with a secondary antibody Horse Radish peroxidase Envision Kit (DAKO) for 20 min and then washed out and incubated with diaminobenzidine for 15 min. Then, after, the slides were washed with PBS, counter-stained with hematoxylin, rehydrated, cleared in xylene, and finally investigated using light microscopy.

#### Evaluation of immunohistochemical results “area %” (specific area/ antibody)

The areas that displayed positive brown immunostaining were selected for evaluation regardless of the strength of staining using some features (cell counter/color deconvolution/color threshold/IHC plugin) of the Image J program. The measurement units (pixels) produced by the Image J program were converted into actual micrometre units. ERα, PR, and VEGF-A immunostaining were measured as area (%) in a standard measuring frame in representative five fields for each subject (ovary and uterus) in all groups using 400× magnification power via light microscopy transferred to the screen.

### Statistical analysis

All data were initially checked for normality, expressed as means and standard error of the mean, and analyzed using SPSS (version 20) using Student’s t-test to compare the FP and LP. *P* values < 0.05 indicated significant differences.

## Results

### Anatomical findings

**Fig. 4:**
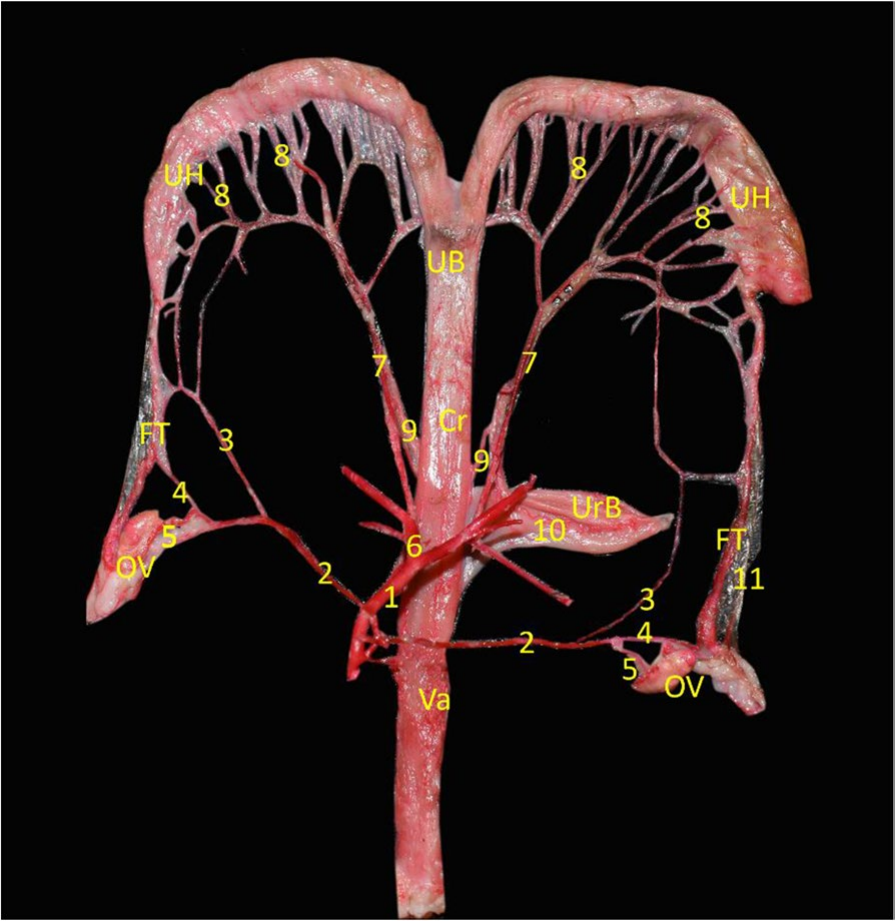
Anatomical picture demonstrated ovary (OV), fallopian tube (FT), uterine horn (UH), uterine body (UB),cervix (Cr), vagina (Va), and urinary bladder (UrB).1- Abdominal aorta, 2-Ovarian artery, 3- Uterine branch of ovarian artery, 4- Tubal branch of ovarian artery, 5- Ovarian branch of ovarian artery, 6- Common iliac artery, 7- Uterine artery, 8- Branches of uterine artery, 9-Vaginal branch of uterine artery, 10 – Cr vesicular artery, 11-Mesosalphnix

The Ovarian (OA) and Uterine (UA) arteries were the main arterial supplies of the genital system. The OA emanated from the abdominal aorta that extended from both sides laterally in a straight direction till released the uterine branch toward the uterine horn. Then, the OA bifurcated into the tubal branch to supply the oviduct and ovarian branch that entered the ovary as the luteal branch from its ventral surface (Fig. 4/ 2). Conversely, the UA was originated from the common iliac artery, passed caudally to ramify and arborize on the uterine horns and uterine body by several minute uterine branches, and provided a communicating branch to the oviduct (Fig. 4/ 7).

### Hemodynamic variations during FP and LP

The OA, LA, and UA cross-sectional diameters (mm) were not affected during the FP and LP. Both Doppler indices (RI and PI) of the OA significantly (*P* < 0.05) increased in the LP compared with those in the FP, reflecting a significant (*P* < 0.05) decrease in the PSV (cm/s) (14.22 ± 0.89 vs. 16.58 ± 0.69), since the PSV of the artery is inversely related with both Doppler indices (Table 1). LA Doppler indices significantly (*P* < 0.05) decreased in the LP compared with those in the FP, suggesting that the LA PSV had a significant (*P* < 0.05) increase (16.25 ± 0.01 vs. 13.65 ± 0.01) (Table 1).

**Table 1:** Doppler scanning during the follicular (Day − 1) and luteal phases (Day 9) in the adult cyclic female rabbits. Values are expressed as mean ± SEM

Variable	Follicular phase Day − 1 (***n*** = 7)	Luteal phase Day 9 (***n*** = 7)	***P***-value
**Ovarian artery (OA)**			
PI	1.32 ± 0.01^b^	1.54 ± 0.02^a^	0.02
RI	0.52 ± 0.02^b^	0.78 ± 0.02^a^	0.02
PSV cm/sec	16.58 ± 0.69^a^	14.22 ± 0.89^b^	0.01
Cross-sectional diameter/mm	4.12 ± 0.06^a^	4.22 ± 0.77^a^	0.88
**Luteal artery (LA)**			
PI	1.25 ± 0.02^a^	0.99 ± 0.01^b^	0.04
RI	0.66 ± 0.01^a^	0.48 ± 0.01^b^	0.01
PSV cm/sec	13.65 ± 0.01^b^	16.25 ± 0.01^a^	0.03
Cross-sectional diameter/mm	3.33 ± 0.46^a^	3.38 ± 0.65^a^	0.13
**Uterine artery (UA)**			
PI	1.88 ± 0.01^a^	1.37 ± 0.01^b^	0.01
RI	0.88 ± 0.01^a^	0.43 ± 0.02^b^	0.01
PSV cm/sec	14.32 ± 0.01^b^	17.35 ± 2.55^a^	0.01
Cross-sectional diameter/mm	4.66 ± 0.08^a^	4.89 ± 0.32^a^	0.25

Values are expressed as mean ± SEM (*n* = 14)

*PI* Pulsatility index, *RI* Resistive index, *PSV* Peak systolic velocity

^a,b^ means with different letters within a row are different (*P* < 0.05)

Finally, Doppler indices in the UA significantly (*P* < 0.05) decreased in the LP compared with those in the FP, whereas those of the LA PSV showed a significant (*P* < 0.05) increase (17.35 ± 2.55 vs. 14.32 ± 0.01) (Table 1).

### Hormonal variations during FP and LP

NO, VEGF, and ILGF were not affected by the phase in the female rabbits (Table 2). However, estradiol (E2) levels showed a marked (*P* < 0.05) decrease in the LP (65.21 ± 2.74) compared with those in the FP (126.28 ± 3.65). Furthermore, progesterone (P4) levels significantly increased (0.36 ± 0.01) in the mid-LP (4.74 ± 0.01) compared with those in the FP (0.36 ± 0.01).

**Table 2:** Hormonal concentrations during the follicular (Day −1) and luteal phases (Day 9) in the adult cyclic female rabbits. Values are expressed as mean ± SEM

Variable	Follicular phase Day − 1 (***n*** = 7)	Luteal phase Day 9 (***n*** = 7)	***P***-value
**Estradiol(E**_**2**_**;pg/mL)**	126.28 ± 3.65^a^	65.21 ± 2.74^b^	0.01
**Progesterone (P**_**4**_**;ng/mL)**	0.36 ± 0.01^b^	4.74 ± 0.01^a^	0.01
**Nitric oxide (NO;μmol/L)**	70.31 ± 10.32^a^	67.82 ± 6.85^a^	0.41
**Vascular endothelial growth factor (VEGF-A; pg/mL)**	51.25 ± 2.22^a^	55.11 ± 6.58^a^	0.22
**Insulin like growth factor (ILGF; ng/mL)**	123.45 ± 2278^a^	136.02 ± 21.35^a^	0.36

^a,b^ means with different letters within a row are different (*P* < 0.05)

### Histological evaluations

#### Light microscopy

**Fig. 5:**
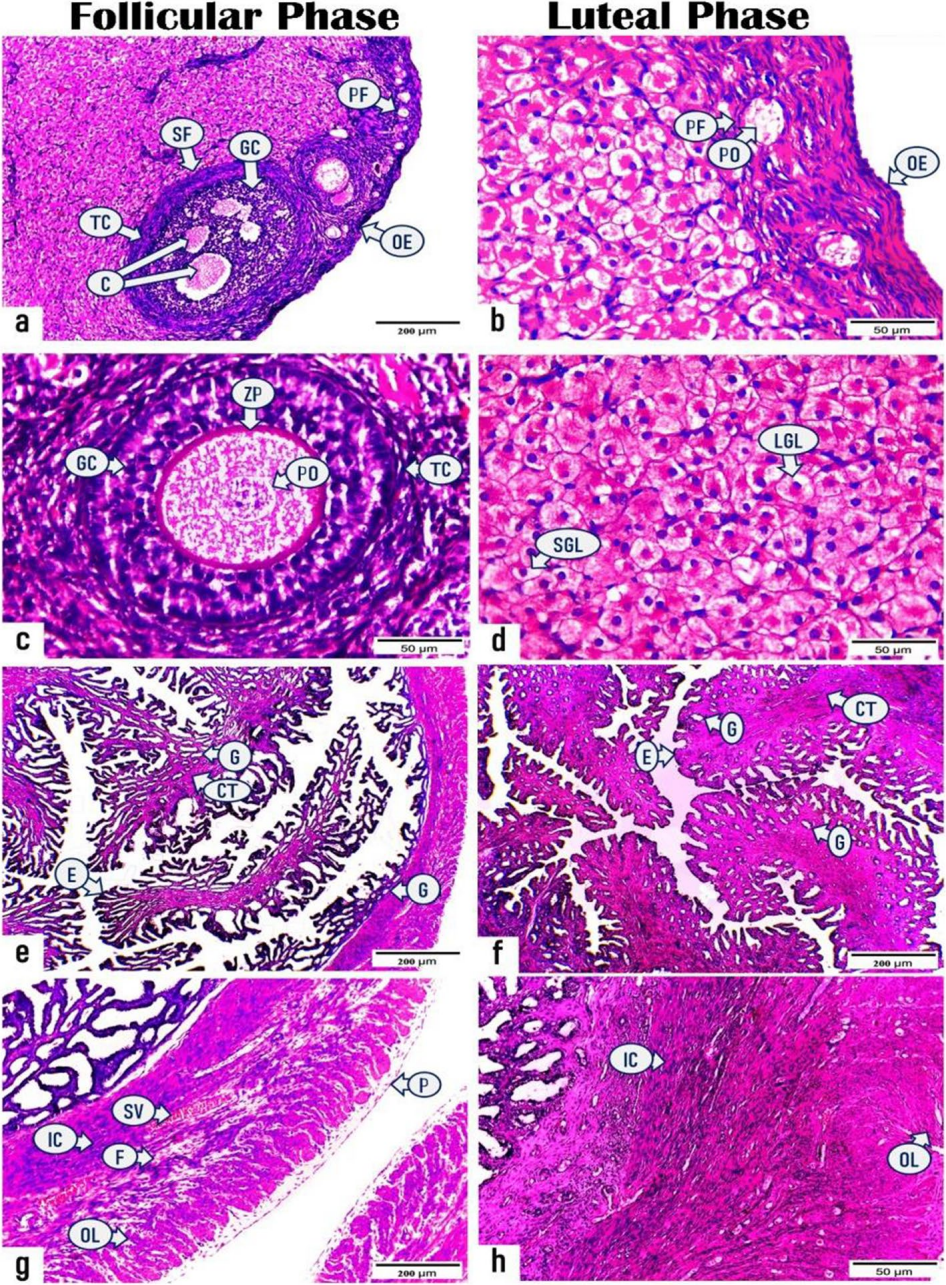
Photomicrographs representing the histological structures of ovarian and uterine sections of rabbits in the follicular and luteal phases. (**a** & **c**): ovarian sections in the follicular phase displaying (**a**) ovarian epithelium (OE), primordial follicle (PF), and secondary follicle (SF). The secondary follicle (SF) contains fluid-filled cavities (C) in between granulosa cells (GC) and encircled by theca cells (TC), (**c**) multi-layered primary follicle presents as primary oocyte (PO) surrounded by zona pellucida (ZP), granulosa cells (GC), and theca cells (TC). (**b** & **d**): ovarian sections in the luteal phase exhibiting (**b**) ovarian epithelium (OE), primary oocyte (PO) inside primordial follicle (PF), (**d**) active corpus luteum with abundant small (SGL) and large granulosa lutein cells (LGL). (**e** & **g**) uterine sections in the follicular phase demonstrating (**e**) high folded endometrial epithelium (E), few connective tissues (CT), and endometrial glands (G), (**g**) myometrium comprising inner circular (IC), outer longitudinal (OL) bundles of smooth muscle, fibrous C.T. (F), and stratum vascular (SV) as well as perimetrium (P). (**f** & **h**) uterine sections in the luteal phase displaying (**f**) endometrial epithelium (E), proliferative connective tissue (CT), endometrial glands (G), (**h**) myometrium with inner circular (IC) and outer longitudinal (OL) smooth muscle. (H & E, Photos no. a, e, f, g: X100, Scale bar = 200 μm, Photos no. b, c, d, h: X400, Scale bar = 50 μm)

Histologically, the cortical region of ovarian sections obtained during the FP (Fig. 5a & c) demonstrated various stages of ovarian follicles with interspersed stromal cells in between. The ovarian follicles were primordial; presented in groups of primary oocytes surrounded by single squamous cells and externally by theca folliculi under the covering germinal epithelium and tunica albuginea and single- and multiple-layered primary follicles; primary oocytes were surrounded by single- and multiple-layered follicular (granulosa) cells encapsulated by theca cells, finally, early and late stages of secondary follicles, wherein the primary oocytes were surrounded by several layers of granulosa cells with the presence of multiple spaces filled with fluid and externally by theca cells. These fluid-filled spaces coalesced with each other forming a single large antrum in the late stage of secondary follicles. The H&E-stained ovarian sections, which were obtained during the LP (Fig. 5b & d), exhibited well-developed and highly active corpora lutea with abundant small and large granulosa lutein cells, separated by blood capillaries, and enclosed by a well-vascularized CT capsule. The granulosa lutein cells were ovoid or polygonal in shape and exhibited spherical vesicular eccentric nuclei with well-defined nucleoli and vacuolated eosinophilic cytoplasm.

Additionally, the H&E-stained uterine sections, which were obtained from rabbits during both FP and LP (Fig. 5e-h), displayed the same histological structure as follows: inner endometrial layer (simple columnar luminal epithelium and lamina propria of the CT containing endometrial glands lined by simple columnar epithelium), middle myometrium (inner circular and outer interwoven longitudinal bundles of smooth muscles supported by dense fibrous CT and stratum vascular in between), and outer perimetrium. The most prominent findings were increasing the luminal epithelium folding and endometrial crypts in addition to the endometrial glands were straighter during the FP; however, during the LP, luminal epithelial cell proliferation was inhibited, with a significant increase in the amount of endometrial CT in addition to some endometrial glands that became more coiled and secretory with different lumina sizes**.**

#### Morphometric analysis

Based on our morphometric data analysis in Table 3, the mean length of the uterine endometrium, area % of endometrial CT, number of endometrial glands, and myometrial area % and thickness were significantly (*P* < 0.05) higher during the LP (147.413 ± 7.280, 24.595 ± 1.216, 319.88 ± 13.023, 33.560 ± 2.527, and 201.171 ± 15.158, respectively) than those during the FP (28.810 ± 2.999, 4.830 ± 0.499, 184.13 ± 18.202, 22.392 ± 2.381, and 134.178 ± 14.298, respectively).

**Table 3:** Scoring measurements of H & E- stained uterine sections during follicular and luteal phases. Values are expressed as mean ± SEM

Variable	Follicular phase Day − 1	Luteal phase Day 9	***P***- Value
**Endometrium (Length (μm))**	28.810 ± 2.999^b^	147.413 ± 7.280^a^	0.001
**Endometrial C.T. (Area %)**	4.830 ± 0.499^b^	24.595 ± 1.216^a^	0.001
**NO. of Glands**	184.13 ± 18.202^b^	319.88 ± 13.023^a^	0.001
**Myometrium (Area %)**	22.392 ± 2.381^b^	33.560 ± 2.527^a^	0.007
**Myometrium (Thickness (μm))**	134.178 ± 14.298^b^	201.171 ± 15.158^a^	0.007

^a,b^ means with different letters within a row are different (*P* < 0.05)

#### Immunohistochemistry

**Fig. 6:**
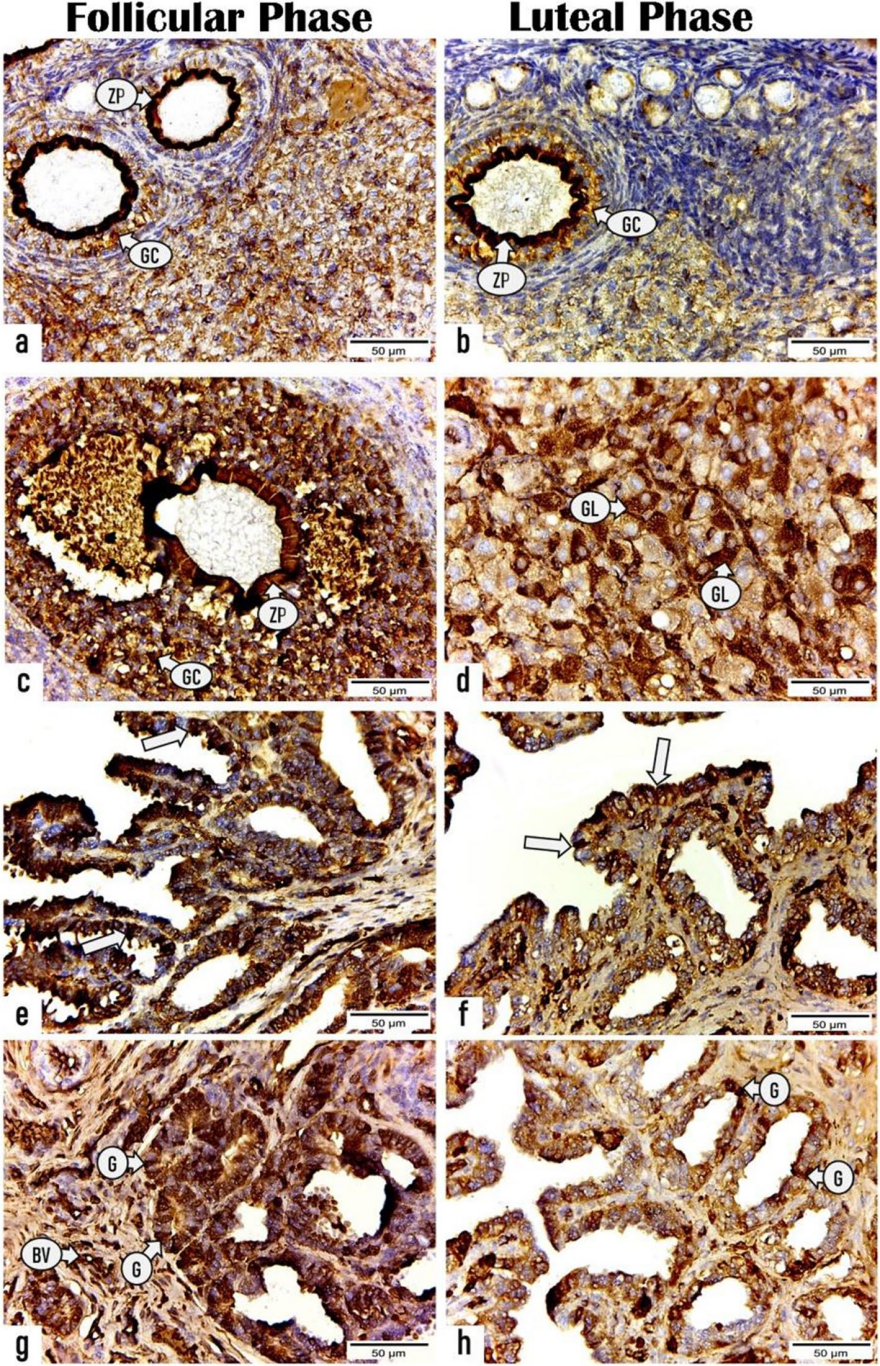
Photomicrographs displaying the immunoexpressing for estrogen receptor in the ovarian and uterine sections of rabbits during the follicular and luteal phases. (a & c): sections of ovary in the follicular phase exhibiting positive ER expression in zona pellucida (ZP) and granulosa cells (GC) of (**a**) primary follicle and (**c**) secondary follicle. (**b** & **d**): sections of ovary in the luteal phase revealing positive reactivity for ER in (**b**) zona pellucida (ZP) and granulosa cells (GC) of primary follicle and (**d**) granulosa lutein cells (GL) of corpus luteum. (**e** & **g**): sections of uterus in the follicular phase showing strong expression for ER receptor in the (**e**) luminal epithelium (arrows), (**g**) endometrial glands (G), and blood vessels (BV). (**f** & **h**) sections of uterus in the luteal phase displaying less reactivity for ER receptor in (**f**) endometrial epithelium (arrows) and (**h**) glands (G). (X400, Scale bar = 50 μm)

**Fig. 7:**
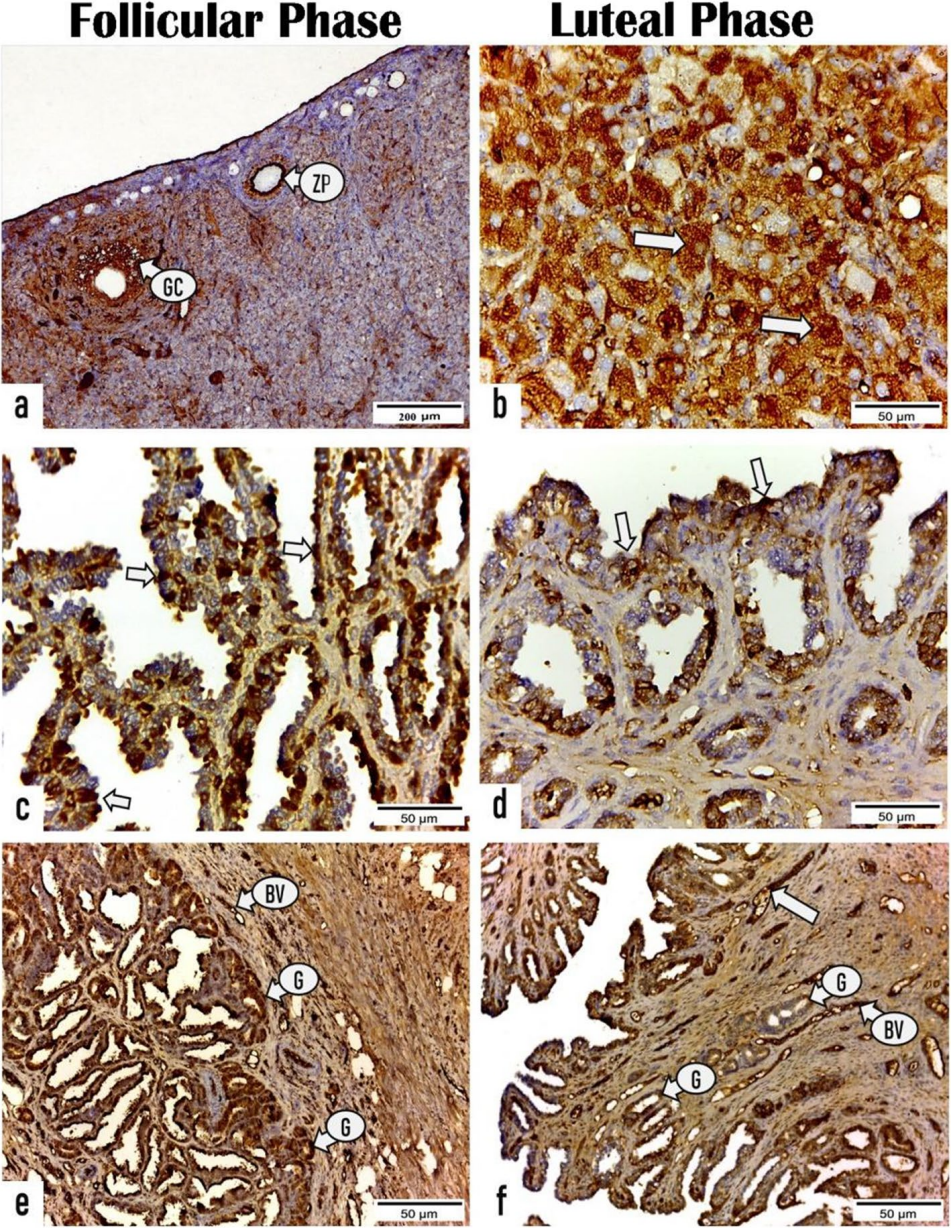
Photomicrographs demonstrating the immunoreactivity for progesterone receptor (PR) in the ovarian and uterine tissue sections of rabbits in the follicular and luteal phases. **a** ovarian section in the follicular phase showing positive PR immune expression in the zona pellucida (ZP) and granulosa cells (GC) of primary follicle. **b** ovarian section in the luteal phase exhibiting positive reactivity for PR in the granulosa lutein cells (arrows) of corpus luteum. **c** & **e** uterine sections in the follicular phase revealing high expression for PR receptor in the (**c**) endometrial epithelium (arrows), (**e**) endometrial glands (G), and blood vessels (BV). **d** & **f** uterine sections in the luteal phase displaying fewer reactivity for PR receptor in the (**d**) endometrial epithelium (arrows), (**h**) glands (G). Notice strong reactivity in the blood vessels (BV). (All photos X400, Scale bar = 50 μm, except photo no. a; X100, Scale bar = 200 μm)

**Fig. 8:**
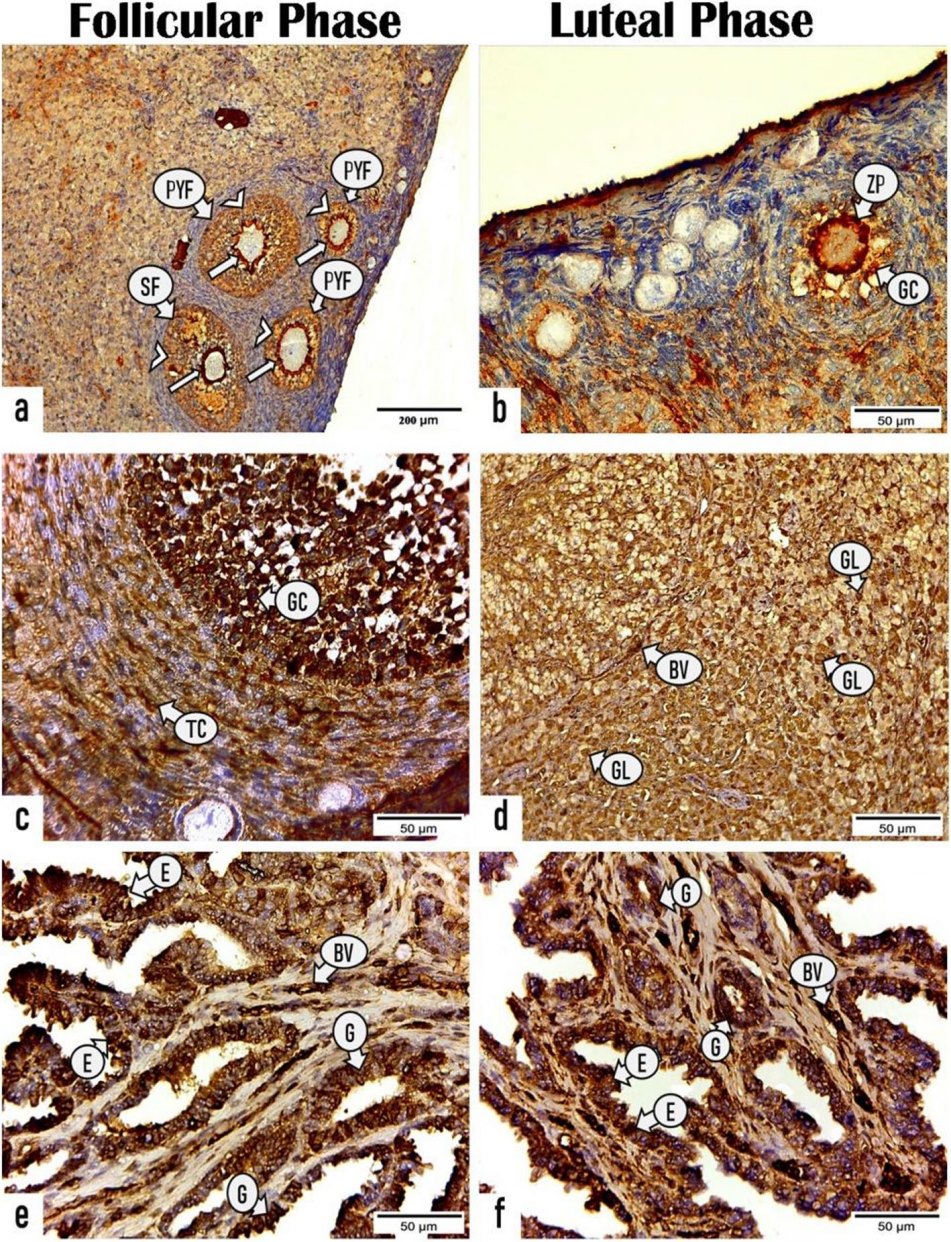
Photomicrographs showing the immunoreactivity for vascular endothelial growth factor (VEGF-A) in the ovarian and uterine tissue sections of rabbits throughout the follicular and luteal phases .**a** & **c** sections of ovary in the follicular phase displaying positive VEGF-A expression in the (**a**) zona pellucida (arrows) and granulosa cells (arrowheads) of primary follicle (PYF) and secondary follicle (SF), (**c**) granulosa cells (GC) and theca cells (TC) of secondary follicle. **b** & **d** sections of ovary in the luteal phase demonstrating positive reactivity for VEGF-A in the (**b**) zona pellucida (ZP) and granulosa cells (GC) of primary follicle and (**d**) granulosa lutein cells (GL) and blood vessels (BV) of the corpora luteum. (e & f): section of uterus in the (**e**) follicular phase and (**f**) luteal phase revealing positive expression for VEGF-A in the luminal epithelium (E), endometrial glands (G), and blood vessels (BV). (All photos: X400, Scale bar = 50 μm, except photo no. a; X100, Scale bar = 200 μm)

The ERs, PRs, and VEGF-A localization was determined immunohistochemically in the ovarian and uterine sections of rabbits during the FP and LP (Figs. 6, 7, 8 and Tables 4, 5).

**Table 4:** Area % of immunostaining scores for estrogen receptor, progesterone receptor, and vascular endothelial growth factor in ovarian sections. Values are expressed as mean ± SEM

Variable		Follicular Phase	Luteal Phase	***P***- Value
**E.R**	a. **O.F.**	43.693 ± 1.349^a^	31.11 ± 0.802^b^	0.001
	b. **C.L.**	7.241 ± 0.572^b^	40.372 ± 0.78^a^	0.001
**P.R.**	a. **O.F.**	17.078 ± 0.70^b^	24.119 ± 0.729^a^	0.001
	b. **C.L.**	25.374 ± 0.87^b^	70.158 ± 0.659^a^	0.001
**VEGFA**A.	a. **O.F.**	27.501 ± 1.298^a^	20.99 ± 0.655^b^	0.001
	b. **C.L.**	26.184 ± 0.934^b^	48.618 ± 0.935^a^	0.001

*ER* Estrogen Receptor, *PR* Progesterone Receptor, *VEGFA* Vascular Endothelial Growth Factor, *O.F* Ovarian Follicles, *CL* Corpus luteum

^a,b^ means with different letters within a row are different (*P* < 0.05)

**Table 5:** Area % of immunostaining scores for estrogen receptor, progesterone receptor, and vascular endothelial growth factor in uterine tissue sections. Values are expressed as mean ± SEM

Variable		Follicular Phase	Luteal Phase	***P***- Value
**Estrogen Receptor**				
I. Endometrium	a. Epithelium	56.650 ± 2.171^a^	20.602 ± 1.910^b^	0.0001
	b. Gland	24.973 ± 1.328^a^	16.170 ± 2.711^b^	0.043
**Progesterone Receptor**				
I. Endometrium	a. Epithelium	44.574 ± 2.434^a^	35.019 ± 2.826^b^	0.043
	b. Gland	47.761 ± 1.841^a^	33.198 ± 2.215^b^	0.001
**Vascular Endothelial Growth Factor**				
I. EndometriumA.	a. Epithelium	48.890 ± 1.673^a^	48.879 ± 0.457^a^	0.995
	b. Gland	49.178 ± 1.642^a^	50.321 ± 2.735^a^	0.732
III. No. of stained capillaries		61 ± 6.557^b^	114 ± 5.291^a^	0.003

^a,b^ means with different letters within a row are different (*P* < 0.05)

##### Ovarian sections

During the FP, both primary and secondary follicles (zona pellucida and granulosa cells) displayed a strong positive immunoreactivity for ERs and VEGF-A with a mean area % of 43.693 ± 1.349 and 27.501 ± 1.298, respectively that are markedly (P < 0.001) higher than those of primary follicles during the LP (31.11 ± 0.802 and 20.99 ± 0.655, respectively) as presented in (Figs. 6, 8 and Table 4). Whereas a significant (*P* < 0.001) immune expression for PRs was observed in the primary follicles during LP with a mean area% of 24.119 ± 0.729 than that during FP 17.078 ± 0.70 as presented in Fig. 7 and Table 4.

Regarding the CL, a substantial (*P* < 0.001) high immunoreactivity for ERs, PRs and VEGF-A was noticed in the granulosa lutein cells of mature CL throughout LP with mean area% of 40.372 ± 0.78, 70.158 ± 0.659, 48.618 ± 0.935, respectively than those of regressed CL during FP (7.241 ± 0.572, 25.374 ± 0.87, 26.184 ± 0.934, respectively) as presented in (Figs. 6, 7, 8 and Table 4).

##### Uterine sections

As shown in (Figs. 6, 7 and Table 5), a substantial greater intensity was observed in the luminal and glandular epithelia during the FP with a mean area % of 56.650 ± 2.171 and 24.973 ± 1.328, respectively for ERs and 44.574 ± 2.434 and 47.761 ± 1.841, respectively, for PRs than those during the LP (20.602 ± 1.910 and 16.170 ± 2.711, respectively for ERs and 35.019 ± 2.826 and 33.198 ± 2.215, respectively for PRs). Furthermore, the endothelium of blood capillaries in the endometrial layer and larger vessels in the stratum vascular of the myometrium revealed an intense positive reactivity for ERs during the FP. While the area % of the immunoreactivity for VEGF-A in the luminal epithelial cells and endometrial glands during the FP (48.890 ± 1.673 and 49.178 ± 1.642, respectively) revealed nonsignificant differences compared with those during the LP (48.879 ± 0.457 and 50.321 ± 2.735, respectively). However, the number of VEGF-A–stained blood capillaries revealed a substantial increase during the LP (114 ± 5.291) compared with that during the FP (61 ± 6.557) as presented in (Fig. 8 and Table 5).

## Discussion

The rabbit ovary was compact and elliptical in its outline, which hung laterally by the mesovarium, as previously described [42]. The right OA emanated before the left one from the abdominal aorta, which was verified in rabbits [43], and chinchilla [44]. Our finding asserted that the OA releases uterine, tubal, and ovarian branches, which was confirmed by Kigata and Shibata [45]; however, this is inconsistent with the finding of Milanović et al. [43]. Therefore, the OA is responsible for ovarian, oviduct, ovarian bursa, and uterine horn nutrition in rabbits [43], and bitch [46]. The UA originated from the common iliac artery, which was inconsistent with the findings of Kigata and Shibata [45], who asserted that it emanated from the umbilical or common iliac artery. The UA is ramified to provide vaginal, cranial vesicular artery, and uterine branches, which is consistent with the findings of Kigata and Shibata [45].

Understanding the does reproductive hemodynamic changes during the estrous cycle is crucial for improving reproductive competence and fertility potential. Based on our results, the Doppler indices (RI and PI) were significantly lower during the FP than those during the LP.

The decreased Doppler indices indicate a decrease in arterial blood flow impedance with subsequent higher ovarian blood perfusion. These results are consistent with those reported in cows [47], buffaloes [48], jennies [49], dromedary camels [50], and women [51]. The mechanisms describing the ovarian hemodynamics improvement, the day before the presumed ovulation, perhaps due to the neo-angiogenesis, arterio-venous connection formations, and vasoactive mediator release, are needed for ovulation and CL formation. Blood capillaries have been found in the granulosa-thecal cells during the LH increase [52] and surge [53].

Furthermore, red blood cells have been demonstrated in the preovulatory human follicles granulosa cell layer on histologic evaluation through the time between LH peak and assumed ovulation [54]. The control of ovarian hemodynamics is governed by steroid hormones, and its exogenous administration triggered substantial changes in the ovarian hemodynamics [55]. The higher estradiol concentration in the FP, as described in this study, may explain the increase in ovarian blood perfusion. Recent studies reported that a strong correlation exists between estradiol (E2), a potent vasodilator, and ovarian blood flow (OBF) [47, 56]. Moreover, an OBF increase was noted after the treatment of postpartum ewes with estradiol 17β [57] . Another possible explanation for the increased OBF is the LH-mediated histamine [58] and/or prostaglandin [59] release that directly affected the vascular permeability, subsequently leading to follicular edema.

Our results showed that the PSV increase and the RI and PI decrease in the UA were higher during the LP than those during the FP. The lower RI and PI values in the LP indicated a higher uterine arterial blood supply [60]. Several studies on cows [47], dromedary camels [50], buffaloes [48], sows [61], and women [62] are consistent with the findings of the current study. Uterine blood flow (UBF) regulations may be explained in the following points: (1) UBF may be increased via acetylcholine-mediated NO (potent vasodilator) synthesis, which modulates the vascular tone [63]; (2) other vasoactive mediators, including vasoactive intestinal polypeptide [64] endothelium-derived hyperpolarization factor [65], and prostacyclin [66], contribute to higher vascularization; and (3) UBF is governed by steroid hormones [67]. In rabbits, both estradiol and PRs were demonstrated in the smooth muscles of the UAs [68], suggesting that steroid hormones, especially estradiol, play a substantial role in UBF regulation [57, 69]. To verify the vasodilator and luteotrophic effect of E2 on the luteal (LBF) and UBF in rabbits, previous studies claimed that decreased E2 in X-ray-damaged ovarian follicles, although not CL, diminished the luteal function, progesterone secretion, and UBF [70].

Regarding the LBF changes in this study, a marked increase in PSV and decrease in RI and PI, which is meant by the CL hypervascularization, were noted during the LP; these values were lower than those in the FP. These results are consistent with those reported in cows [47], dromedary camels [50], and women [62]. The understanding of how the LBF increases depends on several theories. First, the rabbit CL is structurally changed in the LP via the increase in the capillary intensity [71]. Second, the rabbit CL vascularity is sinusoidal capillaries (characterized by the lack of autonomic nerves and smooth muscles in its wall), thereby explaining the low vascular impedance to the blood flow in the CL. Third, the peripheral vasodilatation of the CL via local NO (as reported in the current study) released from the vascular endothelium under the effect of LH-mediated eNOS action [72]; another vasodilatory effect of estradiol was demonstrated by [71], who reported that follicular-based estradiol is substantial for the luteal function of pregnant rabbits. Finally, other vasoactive compounds, including endothelin-1 and angiotensin, may be integrated into the LBF regulation [73, 74].

In this study, the histological investigation of ovarian sections during the FP showed several stages of ovarian follicles during folliculogenisis, which is consistent with the findings of Al-Saffar and Almayahi [5]. Active CL was the most prominent finding in ovarian sections during the LP, which is due to the hyperplasia and hypertrophy of granulosa cells of the ruptured secondary ovarian follicle at the late stage. Furthermore, the presence of primordial and primary ovarian follicles during the LP was evident, which may be due to reduced LH levels and increased progesterone levels during this phase [75]. Moreover, the histological examination of uterine sections at both the FP and LP revealed normal uterine wall architecture, including the endometrium, myometrium, and perimetrium, which is consistent with the findings of [76].

The endometrium is a highly dynamic tissue that undergoes several steroid hormone-induced morphological and functional changes during the estrous cycle and pregnancy [77, 78]. In this study, the endometrium displayed evident morphological and functional changes owing to hormonal status. Based on our results, the FP revealed an increase in the luminal epithelial folding and endometrial crypts; however, the significant (*P* < 0.05) increase in the mean area % of endometrial CT and the number of endometrial glands was particularly observed during the LP. These results may be attributed to the high estrogen levels during the FP that stimulate the lamina epithelialis and upper stromal cells (outer functional zone of the endometrium) proliferation, whereas the increased P4 concentrations during the LP inhibit this proliferation and induce several morphological and functional changes to establish glandular secretory epithelial cells and vascular stroma in addition to increasing stromal cell differentiation [77]. Furthermore, Das et al. [79] stated that the uterine tissue during the interval when the environment moves from the estrous phase (estrogen only) to days 3.5–4 post-ovulation (higher progesterone levels), endometrial cells undergo rapid proliferation, and the increasing number of cells is accommodated by multiple endometrial fold formation. Conversely, on days 4–7 post-ovulation with increasing progesterone levels, epithelial cells undergo differentiation, including the appearance of stage-specific uterine secretions and epithelial cell surface modifications. These changes led to a division of early pseudopregnancy or pregnancy in rabbits into endometrial stages roughly equivalent to the proliferative and secretory phases of the primate cycle, days 1–4 and 4–7, respectively. Thus, the remarkable (*P*< 0.001) increase in the area % of endometrial CT during the LP may be attributed to the increased endometrial gland secretory activity, stromal differentiation, and stromal CT edema under the effect of progesterone [80].

In this study, the ovarian follicles revealed a positive immunoreactivity for ERα and PR during both the FP and LP, which is in harmony with the results in buffaloes [81]. Furthermore, the CL displayed positive immunoreactivity for ERα, which is consistent with the findings of [82] in the cows. Conversely to our result, Pathak et al. [81] noticed weakly or absent ERα immune reaction in the CL. Rosenfeld et al. [82] suggested that the presence of ERα and ERβ mRNA in the corpora lutea is indicative of their involvement in the CL development and maintenance.

Moreover, our study demonstrated a positive immunoreactivity for VEGF-A in the ovarian follicles and CL during both the FP and LP, which is consistent with the findings of [83] who observed strong immunoreactivity for VEGF-A in the tertiary/Graafian follicles and the postovulatory follicular wall of the mare ovary in addition to moderate-to-strong reactivity in the lutein cells of mature CL. These results confirm the significant role of VEGF in intra-follicular oxygenation, dominant follicle selection, and CL development and maintenance [18, 84] providing substrates, including gonadotropins for ovarian follicles and progesterone precursors for CL, and facilitating steroid hormone delivery into the blood circulation [85]. Several studies showed the fundamental role of VEGFs and angiopoietins in mediating ovarian angiogenesis and follicular vascularization in different species, including women [86], cows [87, 88], and mares [85]. Additionally, VEGF is responsible for the increased vascular permeability that enables nutrients, oxygen, and regulatory molecules to supply to the oocyte [89, 90].

Based on our results, the immunohistochemical studies demonstrated that the distribution and intensity of immunoreactivity (staining) for ERs, PRs, and VEGF-A in the different uterine layers alter cyclically during the reproductive cycle in relation to circulating steroid hormone levels. These findings suggest the fundamental role of these receptors in rabbit reproduction. Similar results were reported for cows [91], and ewes [92].

Since the endometrium is the main target for the steroid hormones [93], estrogen in conjunction with progesterone play a significant role in controlling the uterine function mediated by their binding with their specific receptors [94]. In this study, the area % of ER and PR expression in the luminal epithelium and endometrial glands are significantly higher during the FP than that during the LP. These findings are consistent with the results reported in cows [95], in ewes [96], in women [97], and in buffaloes [28]. These results may be attributed to the remarkable increase in the estradiol level during the FP as shown in the current study, which up-regulates the PR and ER immune expression, and the significant increase in the progesterone concentrations produced by the developed CL during the LP down-regulates both receptors [98]. Pathak et al. [28] demonstrated that the lamina epithelialis of a buffalo’s uterus revealed high number of both ERα- and PR-positive cells during the FP compared with that during the LP, whereas the endometrial glands exhibited a significantly (*P* < 0.05) high number of ERα-positive cells and no significantly increased number of PR-positive cells during the FP compared with those during the LP. Winuthayanon et al. [99] showed that ERα is required for uterine epithelium proliferation, consequently preventing its apoptosis.Furthermore, in this study, both luminal and glandular epithelial cells showed positive reactivity for VEGF-A with no significant differences in the area % of immunostaining during both the FP and LP. This result is consistent with the results of Sag˘söz and Saruhan [17] who stated that no significant difference was noted in the intensity of VEGF immunoreactivity and the proportion of positive cells in the luminal epithelium, uterine gland, stroma, and smooth muscles of the bovine uterus during the FP and LP. Based on our results, strong immunoreactivity was observed in the vascular endothelium with a remarkable (*p* < 0.003) increase in the number of stained blood capillaries during the LP. Previous studies reported a strong immunoreactivity for VEGF in the endothelium and smooth muscle of blood vessels during the reproductive cycle [17, 100, 101]. This result suggests that VEGF allows angiogenesis and stimulates vascular permeability changes during the sexual cycle by stimulating the proliferation and migration of vascular endothelium and smooth muscle cells. Furthermore, Alan et al. [102] reported that a particular concentration of VEGF is necessary for inhibiting endothelial cell apoptosis. Moreover, Cullinan-Bove and Koos [103] showed that estrogen regulated the VEGF mRNA expression, whereas progesterone increased the VEGF mRNA levels in the uterus [104]. However, the VEGF expression is not clearly understood and requires further studies.

## Conclusion

The current study displayed that estrogen hormone in combination with progesterone play a critical role in the morphological and morphometric changes in the ovarian and uterine tissues of rabbits that are mediated by binding with their receptors. However, the VEGF-A expression and its role require further studies, but its expression suggests that VEGF plays an important role in angiogenesis, intra-follicular oxygenation, as well as the development of CL and maintenance. Moreover, our study provides valuable information about the does reproductive hemodynamic changes, measured by color spectral Doppler, during the estrous cycle in relation to the serum concentrations of progesterone, estradiol, nitric oxide, and insulin-like growth factor-1that is crucial for improving reproductive competence and fertility potential.

## Data Availability

All data collected or analyzed during this study are included in this published paper.
